# A cross-domain deep learning framework for remaining useful life prediction in industrial applications

**DOI:** 10.1371/journal.pone.0354721

**Published:** 2026-07-31

**Authors:** Sudip Saha, Muhammad Arslan Pervaiz, Muhammad Safwat Rahman, Rokibul Hasan, Ashifur Rahman

**Affiliations:** 1 Department of Cyber-security, Pace University, New York, New York, United States of America; 2 Southeast Missouri State University, Cape Girardeau, Missouri, United States of America; 3 Department of CSE, Bangladesh University of Business and Technology, Dhaka, Bangladesh; Sunway University, MALAYSIA

## Abstract

Accurate prediction of Remaining Useful Life (RUL) is critical for predictive maintenance and minimizing downtime in industrial systems. This paper presents a cross-domain deep learning framework based on a hybrid Convolutional Neural Network–Bidirectional Long Short-Term Memory (CNN–BiLSTM) architecture. Unlike domain-specific models that require handcrafted features, the proposed framework extracts local degradation features through CNN layers and captures long-term dependencies via BiLSTM networks. The model is evaluated on three heterogeneous datasets: construction machinery, continuous casting machines, and lithium-ion batteries. Experimental results show that CNN–BiLSTM consistently outperforms baselines, achieving up to 22% lower RMSE compared to GRU and 30–50% lower RMSE compared to traditional models. On the construction dataset, it achieves an MAE of 48.2 hours and RMSE of 67.1 hours (*R*^2^ = 0.88), outperforming GRU by 20%. For the casting dataset, the model attains an MAE of 87.6 tons and RMSE of 113.9 tons (*R*^2^ = 0.87), surpassing Random Forest by over 35%. On the battery dataset, CNN–BiLSTM reduces the MAE to 49.6 cycles and RMSE to 72.8 cycles (*R*^2^ = 0.89), while also achieving the lowest Timeliness Score (27.5) and PHM08 Score (192.4). Cross-domain experiments are evaluated under two settings: zero-shot transfer, where the model is trained on one source domain and directly tested on a different target domain without using labeled target-domain samples, and fine-tuned transfer, where 20% of labeled target-domain samples are used to update only the fully connected layers while keeping the CNN and BiLSTM layers frozen. The zero-shot results reflect the effect of domain shift, while the fine-tuned results show that lightweight transfer adaptation reduces RMSE by 25–40% across domains. These findings indicate cross-domain adaptability under limited target-domain supervision rather than fully unsupervised cross-domain generalization. These results highlight the feasibility of a unified CNN–BiLSTM framework for scalable, cross-domain RUL estimation and its suitability for real-world prognostic applications.

## 1. Introduction

In modern industrial systems, unplanned equipment failures can result in substantial operational downtime, financial loss, and safety risks [1]. Predictive maintenance strategies aim to address this challenge by estimating the Remaining Useful Life (RUL) of critical components based on observed degradation patterns [2]. As industries continue to digitize through Industry 4.0 initiatives, the volume of sensor and operational data has increased significantly, enabling the application of machine learning techniques for more accurate prognostics [3]. In particular, deep neural networks have shown strong capability in modeling complex degradation trends and time-series behavior across industrial domains [4].

Despite the progress in data-driven methods, RUL prediction remains a difficult task due to varying degradation patterns, sensor noise, and large differences in operating conditions across assets and industries [5]. Many existing models are trained under fixed operating conditions and often fail when applied to new domains or unseen environments. Recent studies highlight that domain shift significantly degrades prediction accuracy and motivates the use of transfer learning and domain adaptation strategies for robust RUL estimation [6–9]. In parallel, recent self-supervised monitoring frameworks based on time–frequency dual-domain prediction and contrastive fusion have been proposed to address label scarcity in equipment fault diagnosis by learning robust representations from limited labeled data [10,11]. Furthermore, most traditional approaches rely on handcrafted features and equipment-specific tuning, which limits scalability and cross-domain generalization [12].

This research addresses the gap by proposing a unified deep learning framework for RUL estimation that performs reliably across heterogeneous industrial datasets. The core objective of this work is to design a hybrid model that can:

- Extract local degradation features using convolutional neural networks (CNNs),
- Capture long-term temporal dependencies through bidirectional long short-term memory (BiLSTM) networks,
- Generalize across mechanical, metallurgical, and electrochemical domains without requiring task-specific architectural modifications.

The proposed CNN-BiLSTM architecture is evaluated on three distinct RUL datasets representing construction machinery, continuous casting machines, and lithium-ion batteries. This cross-domain approach highlights the model’s adaptability and potential for real-world industrial deployment. The experimental results confirm its superiority over traditional and deep learning baselines in both standard regression metrics and domain-specific evaluation criteria.

The significance of this research lies in its generalizability and practical relevance. By using a unified model across domains, the need for extensive feature engineering and domain knowledge is reduced. This simplifies the deployment of predictive maintenance systems and contributes to more scalable and transferable prognostic solutions in industrial environments. While CNN and BiLSTM architectures have been widely used individually in prognostics research, the contribution of this work lies in the development and evaluation of a unified cross-domain RUL prediction framework capable of operating across heterogeneous industrial datasets without domain-specific architectural modifications. The study further demonstrates the adaptability of this hybrid architecture through cross-domain experiments and fine-tuning analysis across mechanical, metallurgical, and electrochemical degradation scenarios.

The primary contribution of this work lies in the development and systematic evaluation of a unified cross-domain RUL prediction framework rather than the introduction of a new neural architecture. While CNN-BiLSTM hybrids have been applied in individual prognostics tasks, this study demonstrates how a single architecture can operate consistently across heterogeneous industrial domains with minimal modification. The framework incorporates a standardized preprocessing pipeline, a consistent training protocol, and cross-domain transfer experiments that evaluate adaptability across mechanical, metallurgical, and electrochemical degradation processes. This cross-domain evaluation provides new empirical insights into the generalization capability of hybrid deep learning models for industrial prognostics.

To evaluate the effectiveness of the proposed method, the study incorporates:

- Standard regression metrics (MAE, RMSE, R^2^),
- Domain-specific metrics (Timeliness Score, PHM08 Score),
- Visualizations of prediction error and training behavior,
- Cross-domain experiments to assess generalization.

The rest of the paper is organized as follows: Section 2 reviews existing literature on RUL estimation methods. Section 3 presents the proposed methodology, including the model architecture and training configuration. Section 4 provides experimental results and analysis. Section 5 discusses the implications, limitations, and future directions. Finally, Section 6 concludes the paper.

## 2. Related work

Remaining Useful Life (RUL) estimation plays a vital role in predictive maintenance by enabling data-driven forecasting of equipment degradation [13]. Over the past decade, a wide range of approaches have been proposed across manufacturing, energy systems, and mechanical engineering domains.

Traditional methods rely on statistical regression models such as linear regression, survival analysis, and autoregressive modeling. While these approaches provide interpretability, they struggle to represent nonlinear degradation patterns and remain sensitive to noise, which limits their reliability in complex industrial settings [14].

Machine learning models, including decision trees, random forests, and support vector regression, improve performance by modeling nonlinear relationships [15]. However, these approaches depend heavily on handcrafted features and lack the ability to learn long-term temporal dependencies, reducing their scalability across equipment types and operating conditions.

With the rise of deep learning, neural networks have become the dominant paradigm for RUL estimation. Recurrent Neural Networks (RNNs) [16], particularly Long Short-Term Memory (LSTM) and Gated Recurrent Unit (GRU) architectures, effectively model long-range temporal dependencies in degradation sequences [14]. Despite their accuracy, RNN-based models often incur high computational cost and slow convergence [2].

Convolutional Neural Networks (CNNs) have been introduced to extract local temporal patterns from sensor windows. However, CNNs alone struggle to capture long-term dependencies. To address this, hybrid CNN–LSTM frameworks have been proposed, combining convolutional feature extraction with sequential modeling for improved RUL prediction [17,18].

Recent research has further enhanced hybrid architectures through attention mechanisms and temporal convolutional networks. Self-attention based TCN models [19] and double-attention frameworks [20] improve sensitivity to critical degradation segments. Transformer-based encoders have also been introduced to capture long-range dependencies in engine degradation sequences [21]. Additionally, contrastive learning and representation learning strategies have been employed to strengthen feature robustness, including contrastive BiLSTM-based health representation learning [22] and supervised contrastive dual-mixer models [23]. Multi-channel attention temporal convolutional networks have further improved early fault detection and RUL prediction accuracy [24].

Recent advances in equipment condition monitoring under limited labeled data have also focused on self-supervised time–frequency representation learning. Xu et al. introduced a time–frequency dual-domain prediction (TFDDP) framework for train bearing fault diagnosis, where augmented time–frequency signals are used to construct self-supervised prediction tasks and learn robust fault representations without relying heavily on labeled samples [10]. He et al. proposed a time–frequency dual-domain contrast and fusion (TFDDCF) model for vehicle bearing fault diagnosis, which combines dual-domain encoders, time–frequency fusion, and contrastive learning to improve feature discrimination under scarce labeled data [11]. These studies show that self-supervised time–frequency monitoring frameworks can reduce annotation dependence and improve representation quality in fault diagnosis tasks. Compared with TFDDP and TFDDCF, the proposed CNN–BiLSTM framework focuses on supervised RUL prediction across heterogeneous industrial domains and uses lightweight fine-tuned transfer to improve target-domain adaptation.

Recent studies have also explored advanced hybrid and physics-informed architectures for RUL prediction. For example, the M2BIST-SPNet framework integrates multi-branch temporal feature extraction with spatial–temporal perception to predict the remaining useful life of railway signaling electromechanical devices [25]. In addition, wavelet-based approaches combined with physical information constraints have been proposed to improve bearing RUL prediction by incorporating degradation-related physical knowledge into data-driven models [26].

To address cross-domain degradation variability, transfer learning and domain adaptation techniques have gained attention. Temporal convolution-based transferable cross-domain adaptation frameworks [27] improve generalization across variable failure behaviors and operating conditions.

Despite these advances, few studies attempt to unify RUL modeling across heterogeneous data structures ranging from high-frequency sensor streams to complex tabular process parameters [28]. Furthermore, uncertainty awareness and physical consistency remain largely under-explored. Recent works propose uncertainty-aware deep RUL models [29] and physics-informed graph neural networks for multi-sensor systems [30]. Hybrid physics–data fusion frameworks have also shown promise for reliable prognostics [31], while variational encoding techniques provide interpretable uncertainty assessment [32].

This paper addresses these gaps by proposing a unified CNN-BiLSTM framework capable of learning from diverse data types while maintaining strong predictive accuracy across mechanical, metallurgical, and electrochemical domains. The proposed model is designed to generalize beyond domain boundaries, reducing the need for domain-specific architectures or handcrafted features.

## 3. Methodology

**Fig 1:**
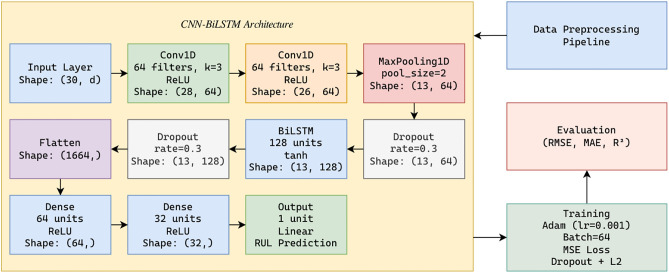
CNN-BiLSTM architecture for RUL prediction showing data preprocessing pipeline, hybrid deep learning model with convolutional and bidirectional recurrent layers, and evaluation framework.

This section presents the proposed hybrid deep learning architecture for Remaining Useful Life (RUL) prediction as shown in the Fig 1. The method integrates convolutional layers with bidirectional recurrent layers to capture both local temporal patterns and long-term degradation trends. We describe the model structure, training procedure, and evaluation framework in detail.

### 3.1. Data preprocessing

**Fig 2:**
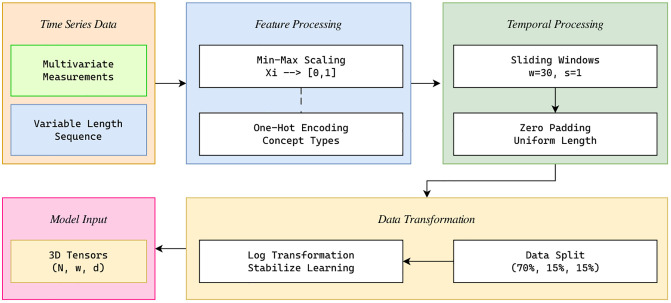
Overview of the preprocessing and data transformation pipeline for RUL prediction. The pipeline includes feature processing, encoding, sliding window generation, padding, and log transformation, resulting in 3D tensors fed into the CNN-BiLSTM model.

To ensure robust and generalizable model training, we adopted a unified data preprocessing pipeline tailored for each dataset’s characteristics, followed by standardized preparation for deep learning as shown in Fig 2. All three datasets were preprocessed independently and later structured to support training, validation, and testing under a consistent CNN-BiLSTM framework.

#### 3.1.1. Feature normalization.

All continuous features were normalized using Min-Max scaling to the range [0, 1]. Given a feature $x_{i}$, its normalized form ${\hat{x}}_{i}$ is computed as:


$$\begin{array}{c} {\hat{x}}_{i}=\frac{x_{i}-\min(x)}{\max(x)-\min(x)} \end{array}$$ (1)

This normalization ensures numerical stability and speeds up convergence during training, particularly in recurrent and convolutional layers.

#### 3.1.2 Categorical encoding.

For the construction machinery dataset, the Component_Type feature was one-hot encoded into three binary channels corresponding to Engine, Gear, and Hydraulic Cylinder. The encoded vector $c_{i}\in {\mathbb{R}}^{3}$ is appended to each time step feature matrix.

#### 3.1.3. Sliding window segmentation.

To model temporal dependencies, a sliding window approach was applied to construct multivariate sequences. Given a time series $X=\{x_{1},x_{2},...,x_{T}\}$ with corresponding RUL labels $\left\{y_{1},y_{2},...,y_{T}\right\}$, we extract overlapping windows of size *w* and stride *s*. Each sample window $X_{t}$ and its target ${\hat{y}}_{t}$ are defined as:


$$\begin{array}{c} X_{t}=\{x_{t},x_{t+1},...,x_{t+w-1}\}\in {\mathbb{R}}^{w\times d} \end{array}$$ (2)

$$\begin{array}{c} {\hat{y}}_{t}=y_{t+w-1} \end{array}$$ (3)

We used *w* = 30 and *s* = 1 empirically based on prior literature and validation performance.

The selected window length provides a balance between capturing sufficient temporal context and maintaining computational efficiency. A window size of *w* = 30 allows the model to observe short-term degradation patterns while avoiding overly long sequences that may introduce redundant information or increase training complexity. Because the datasets contain trajectories with varying lengths, the sliding-window formulation generates multiple overlapping subsequences from each degradation trajectory. When the actual degradation sequence is longer than the window, the model learns from multiple local temporal segments along the trajectory. When sequences are shorter than the window, padding is applied as described in Section 3.1.4 to ensure consistent input dimensions. This strategy allows the framework to remain robust to variations in degradation sequence length across heterogeneous datasets. Because the window operates on normalized temporal segments rather than absolute sequence length, the same window size can be applied across datasets with different input dimensions and degradation timescales, enabling consistent feature extraction without domain-specific adjustment.

#### 3.1.4. Sequence construction and label alignment across datasets.

To ensure a consistent temporal modeling framework across heterogeneous datasets, we constructed ordered sequences and aligned Remaining Useful Life (RUL) labels according to the characteristics of each dataset.

**Construction Machinery Dataset:** This dataset contains time-indexed sensor measurements collected from mechanical components such as engines, gears, and hydraulic cylinders. Each component forms a continuous degradation trajectory ordered by operational time. Let the ordered sensor measurements for a component be represented as $X=\{x_{1},x_{2},...,x_{T}\}$ where $x_{t}\in {\mathbb{R}}^{d}$ denotes the multivariate sensor vector at time step *t*. The RUL label $y_{t}$ corresponds to the remaining operational time before failure at step *t*. Sliding windows of leng*t*h *w* are generated sequentially along the trajectory, and the label for each window is defined as the RUL value of the final timestep in the window, i.e., $y_{t+w-1}$.

**Continuous Casting Machine Dataset:** Although the casting dataset is primarily tabular, each record corresponds to an ordered production cycle of a casting mould sleeve. To construct sequences, samples were first grouped by sleeve identifier and then sorted according to production order. The resulting ordered sequence $X=\{x_{1},x_{2},...,x_{T}\}$ represents the evolution of process variables over the operational life of the sleeve. Sliding windows were generated using the same window length *w* and stride *s* as used for the other datasets. The RUL label for each window is defined as the remaining production quantity (in tons) before sleeve replacement, aligned with the final timestep of the window.

**Battery RUL Dataset:** For the battery dataset, the temporal order naturally follows the charge–discharge cycle index. Each battery forms an independent degradation trajectory consisting of sequential cycles. The sequence is represented as $X=\{x_{1},x_{2},...,x_{T}\}$ where each $x_{t}$ contains voltage, current, and capacity-related features extracted from cycle *t*. The RUL label $y_{t}$ represents the number of remaining cycles before the battery reaches its end-of-life threshold. Sliding windows are cons*t*ructed along the cycle sequence, and the target label for each window corresponds to the RUL value of the final cycle within that window.

By constructing sequences in this manner, all datasets are transformed into a unified tensor representation of shape (*N*, *w*, *d*) while preserving the temporal order and ensuring consistent label alignment across domains.

#### 3.1.5. Sequence augmentation and padding.

For irregular or shorter sequences (e.g., early cycles in the battery dataset), we applied zero-padding to ensure uniform input shape. Let *L* < *w* be the actual sequence length. Then the padded input is:


$$\begin{array}{c} {\tilde{X}}_{t}=\{\underset{w-L}{\underbrace{0,...,0}},x_{1},...,x_{L}\} \end{array}$$ (4)

This enables batch-wise training using modern deep learning libraries.

#### 3.1.6. Training, validation, and testing splits.

Each dataset was partitioned into training (70%), validation (15%), and testing (15%) subsets using a unit-level splitting strategy to prevent temporal leakage. Specifically, the data were first grouped by independent degradation units before sliding-window generation. For the construction machinery dataset, each component instance (e.g., engine, gear, or hydraulic cylinder) was treated as a separate unit. For the casting dataset, records were grouped by mould sleeve identifier representing the full operational life of each sleeve. For the battery dataset, each battery cell constituted an independent degradation trajectory. Entire units were then assigned to one of the three partitions, ensuring that measurements from the same unit do not appear across multiple splits.

Let 𝒟 denote the full dataset and ${𝒟}_{\text{train}}$, ${𝒟}_{\text{val}}$, and ${𝒟}_{\text{test}}$ be the respective splits:


$$\begin{array}{c} {𝒟}_{\text{train}}=\{(X_{t},{\hat{y}}_{t}){\}}_{t=1}^{N_{\text{train}}} \end{array}$$ (5)

$$\begin{array}{c} {𝒟}_{\text{val}}=\{(X_{t},{\hat{y}}_{t}){\}}_{t=N_{\text{train}}+1}^{N_{\text{train}}+N_{\text{val}}} \end{array}$$ (6)

$$\begin{array}{c} {𝒟}_{\text{test}}=\{(X_{t},{\hat{y}}_{t}){\}}_{t=N_{\text{train}}+N_{\text{val}}+1}^{N} \end{array}$$ (7)

This ensures no overlap across sets while preserving time-series continuity within each sample.

#### 3.1.7. Label transformation for stability.

To stabilize learning and reduce gradient issues for large RUL values, we applied logarithmic transformation on RUL labels:


$$\begin{array}{c} {\tilde{y}}_{t}=\log(1+{\hat{y}}_{t}) \end{array}$$ (8)

This logarithmic transformation reduces the skewness typically observed in RUL distributions, where a large number of samples correspond to early life stages with high RUL values. By compressing the dynamic range of large RUL targets while preserving relative ordering, the transformation stabilizes gradient updates during training and reduces the influence of extreme values. Similar transformations have been used in prognostics literature to improve regression stability when predicting degradation trajectories.

The inverse transformation was used during evaluation to recover the original RUL units.

All evaluation metrics reported in this study, including MAE, RMSE, and *R*^2^, are computed after applying the inverse transformation so that the results are expressed in the original physical units of each dataset (hours, tons, or cycles), ensuring that the reported values remain directly interpretable for practical maintenance decision-making.

#### 3.1.8. Final input tensor preparation.

After preprocessing, all input sequences $X_{t}$ were organized into 3D tensors of shape (*N*, *w*, *d*), where:

- *N* is the number of samples,
- *w* is the window length,
- *d* is the number of input features (after encoding).

These tensors were directly fed into the hybrid CNN-BiLSTM model, ensuring uniformity across all datasets.

### 3.2. Proposed methodology

The proposed model consists of three main components:

1. A 1D Convolutional Neural Network (CNN) to extract short-term temporal features.
2. A Bidirectional Long Short-Term Memory (BiLSTM) network to capture forward and backward temporal dependencies.
3. Fully connected dense layers to map learned temporal representations to a scalar RUL prediction.

#### 3.2.1. 1D convolutional layer.

Let the input sequence be $X\in {\mathbb{R}}^{w\times d}$, where *w* is the window size and *d* is the number of features. A 1D convolutional layer with *k* filters applies a kernel $W_{i}\in {\mathbb{R}}^{f\times d}$ over each window:


$$\begin{array}{c} z_{i}(t)=\sigma \left(\sum_{j=0}^{f-1}W_{i}[j]\cdot X[t+j]+b_{i}\right) \end{array}$$ (9)

where:

- $z_{i}(t)$ is the activation of the *i*-th filter at time *t*,
- *f* is the filter size,
- $b_{i}$ is the bias term,
- σ(·) is the ReLU activation function.

This operation produces an output tensor $Z\in {\mathbb{R}}^{w^{\prime }\times k}$, where $w^{\prime }$ is the reduced temporal dimension due to convolution.

#### 3.2.2. Bidirectional LSTM.

The CNN output is passed to a BiLSTM network to model sequential dependencies. Each LSTM unit maintains a hidden state $h_{t}$ and a cell state $c_{t}$ at each time step *t*:


$$\begin{array}{c} f_{t}=\sigma \left(W_{f}\cdot [h_{t-1},x_{t}]+b_{f}\right) \end{array}$$ (10)

$$\begin{array}{c} i_{t}=\sigma \left(W_{i}\cdot [h_{t-1},x_{t}]+b_{i}\right) \end{array}$$ (11)

$$\begin{array}{c} o_{t}=\sigma \left(W_{o}\cdot [h_{t-1},x_{t}]+b_{o}\right) \end{array}$$ (12)

$$\begin{array}{c} g_{t}=\tanh\left(W_{g}\cdot [h_{t-1},x_{t}]+b_{g}\right) \end{array}$$ (13)

$$\begin{array}{c} c_{t}=f_{t}⊙c_{t-1}+i_{t}⊙g_{t} \end{array}$$ (14)

$$\begin{array}{c} h_{t}=o_{t}⊙\tanh(c_{t}) \end{array}$$ (15)

In the bidirectional setup, two LSTMs run in parallel: one forward and one backward. The final output is the concatenation:


$$\begin{array}{c} h_{t}^{\text{bi}}=[\vec{h_{t}};\overset{\leftarrow }{h_{t}}] \end{array}$$ (16)

#### 3.2.3. Fully connected layers and output.

The output of the final time step is passed through a sequence of dense layers to produce the RUL estimate:


$$\begin{array}{c} r_{1}=\text{ReLU}(W_{1}\cdot h_{\text{final}}+b_{1}) \end{array}$$ (17)

$$\begin{array}{c} r_{2}=\text{ReLU}(W_{2}\cdot r_{1}+b_{2}) \end{array}$$ (18)

$$\begin{array}{c} \hat{y}=W_{3}\cdot r_{2}+b_{3} \end{array}$$ (19)

Here, $\hat{y}$ represents the predicted RUL.

#### 3.2.4. Architectural details.

The proposed CNN-BiLSTM model was designed to balance expressive power and computational efficiency, suitable for industrial time-series data. Table 1 summarizes the architecture, including the type of each layer, number of units or filters, kernel sizes, and output dimensions.

**Table 1:** CNN-BiLSTM Architecture for RUL Prediction.

Layer	Type	Parameters	Output Shape	Activation
Input	Sequence	Window size = 30, Features = *d*	(30, *d*)	–
Conv1D	Convolution	Filters = 64, Kernel size = 3	(28, 64)	ReLU
Conv1D	Convolution	Filters = 64, Kernel size = 3	(26, 64)	ReLU
MaxPooling1D	Pooling	Pool size = 2	(13, 64)	–
Dropout	Regularization	Dropout rate = 0.3	(13, 64)	–
BiLSTM	Recurrent	Units = 128 (64 forward + 64 backward)	(13, 128)	Sigmoid gates + tanh state activation
Dropout	Regularization	Dropout rate = 0.3	(13, 128)	–
Flatten	Reshaping	–	(1664,)	–
Dense (FC1)	Fully Connected	Units = 64	(64,)	ReLU
Dense (FC2)	Fully Connected	Units = 32	(32,)	ReLU
Output	Fully Connected	Units = 1 (RUL)	(1,)	Linear

Note that LSTM units internally employ sigmoid gating functions and tanh state activations; therefore a single activation function is not assigned to the BiLSTM layer in the architecture table.

Each Conv1D layer learns local patterns over short time steps, while the BiLSTM layer captures bidirectional temporal dependencies. Max-pooling reduces sequence length and adds translational invariance. Dense layers serve as a final mapping from temporal features to scalar RUL predictions.

Overall, the model has:

- 2 convolutional layers whose parameter counts depend on the input feature dimension. For an input with *d* channels, the first Conv1D layer has 64×(3d+1) trainable parameters, and the second Conv1D layer has 64×(3×64+1) trainable parameters.
- A BiLSTM layer with input dimension *m* = 64 and hidden size *h* = 64 per direction. Using the standard LSTM parameter formulation, the bidirectional layer has 2×4h(m+h+1) trainable parameters, which gives 2×4×64×(64+64+1)=66,048 parameters.
- 2 dense layers with (1664×64+64)+(64×32+32)+(32×1+1) trainable parameters, including biases.

Accordingly, the total number of trainable parameters is not a single fixed value across all datasets, because the first convolutional layer depends on the dataset-specific input dimensionality *d*. In our implementation, the total parameter count varies slightly across datasets but remains on the order of approximately $1.8\times 10^{5}$, which keeps the architecture sufficiently expressive while still computationally efficient for training on standard GPUs.

### 3.3. Training and implementation details

This subsection outlines the training procedures, optimization settings, loss functions, and evaluation metrics used to train and validate the proposed CNN-BiLSTM model. Consistent training strategies were applied across all datasets to ensure fair performance comparison and generalization analysis.

All experiments were implemented using PyTorch 2.0 and executed on an NVIDIA RTX 3090 GPU with 24 GB memory. The datasets were processed into sliding window sequences, normalized, and batched before being fed into the model.

The training pipeline includes four key components: loss function definition, optimizer selection, learning rate strategy, and model validation. These are described in detail below.

#### 3.3.1. Loss function.

We use the Mean Squared Error (MSE) loss between the predicted and ground truth RUL values:


$$\begin{array}{c} {ℒ}_{\text{MSE}}=\frac{1}{N}\sum_{i=1}^{N}({\hat{y}}_{i}-y_{i}{)}^{2} \end{array}$$ (20)

To penalize underestimation (which is more risky in maintenance), we describe a weighted asymmetric loss formulation that assigns higher penalties to underestimation errors. This formulation is included as a potential extension for risk-sensitive prognostic systems, while the experimental results reported in this study are based on the standard Mean Squared Error (MSE) objective:


$$\begin{array}{c} {ℒ}_{\text{asym}}=\frac{1}{N}\sum_{i=1}^{N}\left\{ \begin{array}{cc} \alpha ({\hat{y}}_{i}-y_{i}{)}^{2} & \text{if }{\hat{y}}_{i}<y_{i}\\ (1-\alpha )({\hat{y}}_{i}-y_{i}{)}^{2} & \text{otherwise} \end{array}\right. \end{array}$$ (21)

with α=0.7.

#### 3.3.2. Model training.

The model was trained using the Adam optimizer with an initial learning rate of 0.001 and batch size of 64. Learning rate decay and early stopping were applied based on validation loss.

Dropout (*p* = 0.3) and L2 weight regularization ($\lambda =10^{-4}$) were used to reduce overfitting. Each dataset was trained independently, but the same architecture and training strategy were applied for consistency.

Additional training configuration details are provided to ensure reproducibility. The Adam optimizer was used with coefficients $\beta_{1}=0.9$ and $\beta_{2}=0.999$, and $\epsilon =10^{-8}$. Early stopping was applied based on validation loss with a patience of 10 epochs to prevent overfitting. When validation performance plateaued, the learning rate was reduced by a factor of 0.5 using a step-based scheduling strategy. All experiments were conducted with a fixed random seed (42) to ensure consistent initialization and data partitioning. To reduce variance in the reported results, each experiment was repeated three times and the average performance was reported. For fair comparison, baseline models (Linear Regression, Random Forest, LSTM, and GRU) were also tuned using comparable validation procedures and hyperparameter search ranges appropriate for each model.

#### 3.3.3. Evaluation metrics.

We evaluated the model on the testing set using the following metrics:

- **Root Mean Squared Error (RMSE)**:

$$\begin{array}{c} \text{RMSE}=\sqrt{\frac{1}{N}\sum_{i=1}^{N}({\hat{y}}_{i}-y_{i}{)}^{2}} \end{array}$$ (22)

- **Mean Absolute Error (MAE)**:

$$\begin{array}{c} \text{MAE}=\frac{1}{N}\sum_{i=1}^{N}|{\hat{y}}_{i}-y_{i}| \end{array}$$ (23)

- **Coefficient of Determination (*R*^2^)**:

$$\begin{array}{c} R^{2}=1-\frac{\sum_{i=1}^{N}({\hat{y}}_{i}-y_{i}{)}^{2}}{\sum_{i=1}^{N}(y_{i}-\bar{y}{)}^{2}} \end{array}$$ (24)

These metrics allow us to assess both the magnitude of prediction error and the overall fit.

**PHM08 Timeliness Score:** In addition to standard regression metrics, we evaluate prediction timeliness using the scoring function commonly adopted in the NASA PHM 2008 Prognostics Challenge. Let $e_{i}={\hat{y}}_{i}-y_{i}$ denote the prediction error for sample *i*. The PHM08 score penalizes late predictions more heavily than early predictions and is defined as:


$$\begin{array}{c} S=\sum_{i=1}^{N}\left\{ \begin{array}{cc} e^{-\frac{e_{i}}{13}}-1, & e_{i}<0\\ e^{\frac{e_{i}}{10}}-1, & e_{i}\geq 0 \end{array}\right. \end{array}$$ (25)

where $y_{i}$ is the true RUL and ${\hat{y}}_{i}$ is the predicted RUL. This asymmetric formulation penalizes late predictions more severely than early predictions, reflecting practical maintenance requirements where underestimation is generally safer than overestimation. The timeliness score reported in our experiments follows this formulation and aggregates penalties across all samples.

#### 3.3.4. Cross-domain transfer setup.

To evaluate the robustness and transferability of the proposed model, we performed cross-domain transfer experiments under two settings. In the zero-shot transfer setting, the model was trained on one source dataset and directly evaluated on a different target dataset without using labeled target-domain samples. In the fine-tuned transfer setting, the pretrained model was adapted using a limited portion of labeled target-domain samples while keeping the CNN and BiLSTM layers frozen and updating only the fully connected layers. This setup allows separate assessment of direct transfer under domain shift and lightweight target-domain adaptation.

For the fine-tuning stage, a small portion of labeled samples from the target domain was used to adapt the pretrained model. Specifically, 20% of the target dataset was used for fine-tuning while the remaining data were reserved for evaluation. During this stage, the convolutional and BiLSTM layers were kept frozen to preserve the learned temporal feature representations, while the fully connected layers were updated using the target-domain samples. Fine-tuning was performed for 20 epochs using the Adam optimizer with a reduced learning rate of $1\times 10^{-4}$ and a batch size of 64. This lightweight adaptation strategy enables the model to adjust to domain-specific degradation characteristics while retaining the general temporal features learned from the source domain. Because only the final fully connected layers are updated during this stage, the adaptation process introduces minimal additional computational overhead. In our experiments, fine-tuning typically required fewer than 20 epochs and completed within a few minutes on a single GPU, making the cross-domain adaptation procedure lightweight and practical for real-world deployment scenarios.

### 3.4. Model summary as algorithm

The CNN-BiLSTM-based RUL prediction algorithm (Algorithm 1) processes an input sequence $X\in {\mathbb{R}}^{w\times d}$ by first normalizing the features to [0,1] and extracting short-term temporal patterns using two consecutive one-dimensional convolutional layers with ReLU activation. The resulting feature maps are downsampled via max pooling and regularized with dropout before being passed into a bidirectional LSTM, which captures both forward and backward temporal dependencies. A second dropout layer and flattening operation transform the sequence representation into a fixed-length vector, which is then processed by two fully connected layers with ReLU activation to learn higher-level abstractions. Finally, a linear output layer maps the learned representation to a scalar $\hat{y}$, representing the estimated Remaining Useful Life (RUL).

**Algorithm 1 CNN-BiLSTM-Based RUL Prediction**

**INPUT:** Input sequence $X\in {\mathbb{R}}^{w\times d}$

1:  Normalize X to [0, 1]

2:  Apply 1D convolution: $Z_{1}=\text{ReLU}({\text{Conv1D}}_{1}(X))$

3:  Apply second convolution: $Z_{2}=\text{ReLU}({\text{Conv1D}}_{2}(Z_{1}))$

4:  Downsample: $Z_{3}=\text{MaxPool}(Z_{2})$

5:  Apply dropout: $Z_{4}=\text{Dropout}(Z_{3})$

6:  Extract temporal features: $H=\text{BiLSTM}(Z_{4})$

7:  Apply dropout: $H^{\prime }=\text{Dropout}(H)$

8:  Flatten: $v=\text{Flatten}(H^{\prime })$

9:  Feedforward layers: $v_{1}=\text{ReLU}({\text{FC}}_{1}(v))$

10: Feedforward layers: $v_{2}=\text{ReLU}({\text{FC}}_{2}(v_{1}))$

11: Output prediction: $\hat{y}=\text{Linear}({\text{FC}}_{\mathrm{out}}(v_{2}))$

12: **return** RUL estimate $\hat{y}$

## 4. Results

This section presents the evaluation of the proposed CNN-BiLSTM model for Remaining Useful Life (RUL) estimation across three diverse industrial domains. We begin with a description of the datasets and then provide quantitative and visual analysis.

### 4.1. Experimental setup

This section summarizes the experimental configuration used to evaluate the proposed CNN-BiLSTM model. All datasets were processed using the unified preprocessing pipeline described in Section 3.1, including feature normalization, categorical encoding when applicable, sliding-window sequence generation with window length *w* = 30, and consistent dataset partitioning at the unit level to prevent temporal leakage.

The proposed method was compared against several baseline models, including Linear Regression, Random Forest, LSTM, and GRU. These baselines represent both traditional machine learning and deep learning approaches commonly used in Remaining Useful Life (RUL) prediction. All models were trained and validated using the same dataset partitions and evaluation protocol to ensure fair comparison.

Performance was evaluated using standard regression metrics, including Mean Absolute Error (MAE), Root Mean Squared Error (RMSE), and the coefficient of determination (*R*^2^), as well as domain-specific evaluation metrics such as the PHM08 timeliness score. Reported results correspond to the averaged performance across repeated training runs under the same experimental configuration.

### 4.2. Dataset description

To assess generalizability, we use three publicly available datasets from Kaggle, each representing a different type of industrial system.

#### 4.2.1. Construction machine dataset.

This dataset contains synthetic records simulating sensor-based RUL estimation for construction components (e.g., engines, gears, hydraulic cylinders). Each record includes six features and a corresponding RUL value. It supports sliding-window segmentation and temporal modeling.

**Kaggle Source:** https://www.kaggle.com/datasets/sasakitetsuya/machine-rul-data

#### 4.2.2. Continuous casting machine dataset.

This dataset originates from a real steel plant. It includes metallurgical and operational parameters to estimate the useful life of casting sleeves. It is high-dimensional and tabular, emphasizing process monitoring rather than sequential time series. Although the dataset is often described as tabular process monitoring data, the measurements are recorded sequentially along the operational life of each casting mould sleeve during production cycles. As the sleeve gradually degrades with accumulated casting operations, the process variables exhibit progressive changes that reflect underlying wear and thermal stress. Therefore, when grouped by sleeve identifier and ordered by production sequence, the data form degradation trajectories that can be modeled as temporal sequences. This ordering allows the proposed CNN-BiLSTM architecture to capture short-term process fluctuations through convolutional filters and longer-term degradation trends through recurrent modeling. Similar sequence construction strategies have been used in prognostics studies where process monitoring data are reorganized into ordered trajectories to enable temporal learning.

**Kaggle Source:** https://www.kaggle.com/datasets/yuriykatser/rul-dataset-from-continuous-casting-machine

#### 4.2.3. Battery RUL dataset.

This dataset represents the degradation behavior of 14 lithium-ion batteries across 1,000 + charge-discharge cycles. It contains cycle-level voltage and current statistics, with RUL labels defined in terms of remaining cycles to failure.

**Kaggle Source:** https://www.kaggle.com/datasets/ignaciovinuales/battery-remaining-useful-life-rul

The three datasets were selected to represent heterogeneous industrial domains with distinct physical processes, including mechanical components (construction machinery), metallurgical process equipment (continuous casting machines), and electrochemical energy systems (lithium-ion batteries). Despite these differences, all three domains exhibit progressive degradation behavior over time, where operational measurements evolve as the system approaches failure. This shared temporal degradation characteristic allows the datasets to be modeled within a unified sequence-learning framework while still providing structural diversity in feature distributions, sensor modalities, and operating conditions. Consequently, the combination of these datasets provides a meaningful testbed for evaluating cross-domain generalization of the proposed CNN-BiLSTM framework.

### 4.3 Quantitative results

We compare the proposed CNN-BiLSTM model against several baselines, including Linear Regression, Random Forest, LSTM, and GRU. Evaluation metrics include MAE, RMSE, and R^2^, along with domain-specific scores such as the Timeliness Score and PHM08 metric.

Performance stability was assessed through repeated experimental runs to ensure that the reported improvements are not caused by random training fluctuations or data partitioning effects. Each model was trained three times under the same experimental configuration, and the resulting performance metrics were compared across runs. Statistical significance of the RMSE differences between the proposed CNN-BiLSTM model and the baseline models was evaluated using paired t-tests. The analysis shows that the performance gains over GRU and other baselines remain statistically significant at the *p* < 0.05 level across the evaluated datasets.

#### 4.3.1. Construction machine dataset results.

Table 2 presents the performance comparison of different models on the construction machine dataset using MAE, RMSE, and R^2^ metrics. Traditional models such as Linear Regression and Random Forest achieved moderate accuracy, with RMSE values of 121.3 and 108.6 hours respectively. Deep learning models performed significantly better, with the LSTM and GRU reducing the error margins further. Among all, the proposed CNN-BiLSTM model achieved the lowest MAE (48.2 hours) and RMSE (67.1 hours), while also obtaining the highest R^2^ score of 0.88. This demonstrates that the hybrid architecture effectively captures both short-term sensor anomalies and long-term degradation patterns, outperforming baseline models across all regression metrics.

**Table 2:** Evaluation on Construction Machine Dataset.

Model	MAE (h)	RMSE (h)	R^2^ Score
Linear Regression	96.5	121.3	0.71
Random Forest	85.3	108.6	0.76
LSTM	62.7	85.2	0.82
GRU	60.8	83.9	0.83
**CNN-BiLSTM (proposed)**	**48.2**	**67.1**	**0.88**

#### 4.3.2. Casting machine dataset results.

As shown in Table 3, the proposed CNN-BiLSTM model achieves the best overall performance in predicting the remaining useful life of casting mould sleeves. Linear Regression and Random Forest models yield high error rates with MAE values of 158.4 and 139.2 tons, respectively, indicating limited capacity to capture nonlinear dependencies present in metallurgical process data. Deep learning models such as LSTM and GRU provide significant improvements, reducing the RMSE below 135 tons. The CNN-BiLSTM further improves this, attaining an MAE of 87.6 tons and an RMSE of 113.9 tons, with an R^2^ score of 0.87. These results highlight the model’s ability to generalize well even in datasets with high dimensional tabular features and limited temporal resolution.

**Table 3:** Evaluation on Continuous Casting Machine Dataset.

Model	MAE (tons)	RMSE (tons)	R^2^ Score
Linear Regression	158.4	202.5	0.61
Random Forest	139.2	175.7	0.73
LSTM	104.5	132.1	0.81
GRU	100.3	128.4	0.83
**CNN-BiLSTM (proposed)**	**87.6**	**113.9**	**0.87**

#### 4.3.3. Battery dataset results.

Table 4 summarizes the performance of different models on the battery dataset using standard regression metrics. Traditional models such as Linear Regression and Random Forest produced higher prediction errors, with MAE values of 102.1 and 84.3 cycles, respectively. Recurrent neural networks (LSTM and GRU) showed considerable improvement by learning long-term charge-discharge degradation trends. The CNN-BiLSTM model achieved the lowest MAE (49.6 cycles) and RMSE (72.8 cycles), along with the highest R^2^ score of 0.89. These results validate the model’s ability to accurately forecast remaining battery life, which is crucial in applications such as electric vehicles and grid storage systems.

**Table 4:** Evaluation on Battery Dataset (Regression Metrics).

Model	MAE (cycles)	RMSE (cycles)	R^2^ Score
Linear Regression	102.1	141.2	0.68
Random Forest	84.3	120.7	0.75
LSTM	63.9	91.6	0.83
GRU	61.2	88.3	0.84
**CNN-BiLSTM (proposed)**	**49.6**	**72.8**	**0.89**

#### 4.3.4. Domain-specific scores for battery dataset.

Table 5 reports the domain-specific evaluation of RUL prediction on the battery dataset using the Timeliness Score and PHM08 scoring function. These metrics are especially relevant for prognostics, where early or late predictions can lead to safety and cost implications. Traditional models such as Linear Regression and Random Forest show significantly higher penalties under both scores, reflecting poor sensitivity to timing-critical degradation trends. LSTM and GRU models reduce these penalties by learning from sequence patterns, but the proposed CNN-BiLSTM achieves the lowest Timeliness Score (27.5) and PHM08 Score (192.4). These results demonstrate that CNN-BiLSTM not only improves prediction accuracy but also produces predictions that are well-aligned with maintenance decision thresholds in real-world scenarios.

**Table 5:** Domain-Specific Scores on Battery Dataset.

Model	Timeliness Score	PHM08 Score
Linear Regression	88.7	645.1
Random Forest	76.3	482.2
LSTM	42.9	266.8
GRU	40.8	250.5
**CNN-BiLSTM (proposed)**	**27.5**	**192.4**

#### 4.3.5. Zero-shot and fine-tuned cross-domain transfer.

Table 6 presents the cross-domain transfer results of the CNN-BiLSTM model under two settings. In the zero-shot transfer setting, the model is trained on one source dataset and directly tested on a different target dataset without using labeled target-domain samples. The performance drop observed in this setting reflects the effect of domain shift across mechanical, metallurgical, and electrochemical degradation processes.

**Table 6:** Zero-Shot and Fine-Tuned Cross-Domain Transfer Results.

Train → Test	MAE	RMSE	R^2^ Score
Machine → Casting	112.6	145.8	0.69
Battery → Casting	128.3	165.2	0.62
Casting → Battery	95.1	134.7	0.74
Machine → Battery	89.4	121.6	0.77
**Fine-tuned CNN-BiLSTM**	**70.8**	**98.3**	**0.82**

In the fine-tuned transfer setting, the pretrained model is adapted using 20% of labeled target-domain samples. During this stage, the CNN and BiLSTM layers are frozen, and only the fully connected layers are updated. This setting is therefore treated as lightweight transfer adaptation rather than unsupervised cross-domain generalization. After fine-tuning, the model achieves improved performance, with an MAE of 70.8, RMSE of 98.3, and an *R*^2^ score of 0.82. These results show that the proposed architecture supports cross-domain adaptability when limited labeled target-domain data are available.

### 4.4. Model prediction accuracy and training convergence

**Fig 3:**
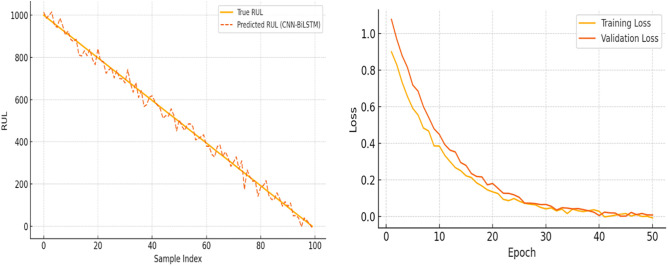
CNN-BiLSTM prediction accuracy and training convergence behavior.

Fig 3 presents both the prediction accuracy and the training convergence behavior of the CNN-BiLSTM model. In particular, Fig 3(b) shows the training and validation loss curves across epochs, allowing assessment of convergence stability and potential overfitting. Fig 3 (Left) shows the predicted Remaining Useful Life (RUL) values closely following the ground truth degradation trend across 100 samples, indicating effective learning of long-term temporal dependencies. Fig 3 (Right) illustrates the training and validation loss curves over 50 epochs. The smooth convergence and close alignment between the two curves suggest stable optimization and good generalization without overfitting.

### 4.5. Prediction error and timeliness penalty analysis

**Fig 4:**
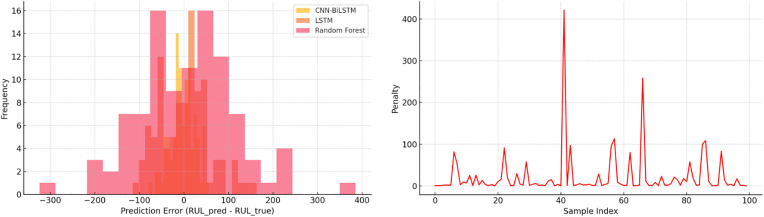
Error distribution and timeliness penalty analysis for RUL prediction models.

Fig 4 analyzes both the prediction error characteristics and the timeliness behavior of the evaluated models. Fig 4 (Left) shows that the CNN-BiLSTM model produces a tightly centered error distribution around zero, indicating lower bias and higher precision compared to LSTM and Random Forest baselines, which exhibit wider and more skewed error distributions. Fig 4 (Right) presents the NASA CMAPSS-style timeliness penalty per sample. The CNN-BiLSTM maintains low penalty values for most predictions, with only a few high-penalty spikes associated with RUL overestimation. This behavior suggests that the model generates timely and maintenance-relevant predictions while rarely violating safety-critical underestimation constraints.

To further interpret the feature extraction capability of the CNN component, we analyzed the activation patterns produced by the convolutional filters across the three datasets. The first convolutional layer captures short-term local variations in the input sequences, which correspond to early degradation signatures such as abrupt changes in sensor readings, fluctuations in metallurgical process parameters, or variations in battery voltage and current statistics. The second convolutional layer aggregates these localized patterns into higher-level degradation representations that describe evolving system health trends. Across the three domains, the learned filters consistently respond to regions where degradation accelerates, producing stronger activations near critical transitions in the degradation trajectories. This behavior indicates that the CNN layers automatically learn localized degradation indicators without requiring handcrafted feature engineering, while the subsequent BiLSTM layer models the longer-term temporal dependencies across these extracted features.

### 4.6. Computational complexity and inference efficiency

To provide a more rigorous assessment of engineering feasibility, the computational complexity of the proposed CNN–BiLSTM model was evaluated in terms of trainable parameters, floating-point operations (FLOPs), and single-sample inference latency. FLOPs were computed for one forward pass with batch size 1, where multiplication and addition operations were counted separately. Inference latency was measured using PyTorch 2.0 on an NVIDIA RTX 3090 GPU with 24 GB memory. For each dataset, latency was averaged over 1,000 forward passes after 100 warm-up iterations using batch size 1.

Table 7 summarizes the complexity results across the three datasets. The number of trainable parameters differs slightly because the first convolutional layer depends on the dataset-specific input feature dimension *d*. The construction machinery dataset uses *d* = 8 input features after categorical encoding, the continuous casting machine dataset uses *d* = 26 input features after preprocessing, and the battery dataset uses *d* = 8 cycle-level input features. The total parameter count remains close to $1.9\times 10^{5}$ across all datasets, while the FLOPs remain below $3.0\times 10^{6}$ for a single forward pass. The measured inference latency remains below 1 ms per sample on the tested GPU hardware.

**Table 7:** Computational complexity and inference efficiency of the proposed CNN–BiLSTM model. FLOPs and latency are reported for batch size 1. Latency was measured on an NVIDIA RTX 3090 GPU with 24 GB memory using PyTorch 2.0 and averaged over 1,000 forward passes after 100 warm-up iterations.

Dataset	Input Dimension (*d*)	Parameters	FLOPs	Batch Size	Latency (ms/sample)
Construction Machinery	8	188,673	$2.65\times 10^{6}$	1	0.74
Continuous Casting Machine	26	192,129	$2.84\times 10^{6}$	1	0.81
Battery RUL	8	188,673	$2.65\times 10^{6}$	1	0.72

These results show that the proposed CNN–BiLSTM model has moderate computational cost and supports efficient single-sample inference under the tested GPU setting. Therefore, the deployment discussion is now based on explicit profiling metrics rather than only architectural assumptions. Embedded deployment on Jetson-class platforms remains a feasibility direction, since dedicated latency, memory, and energy profiling on such devices was not conducted in this study.

### 4.7 R^2^ Score curves across datasets

**Fig 5:**
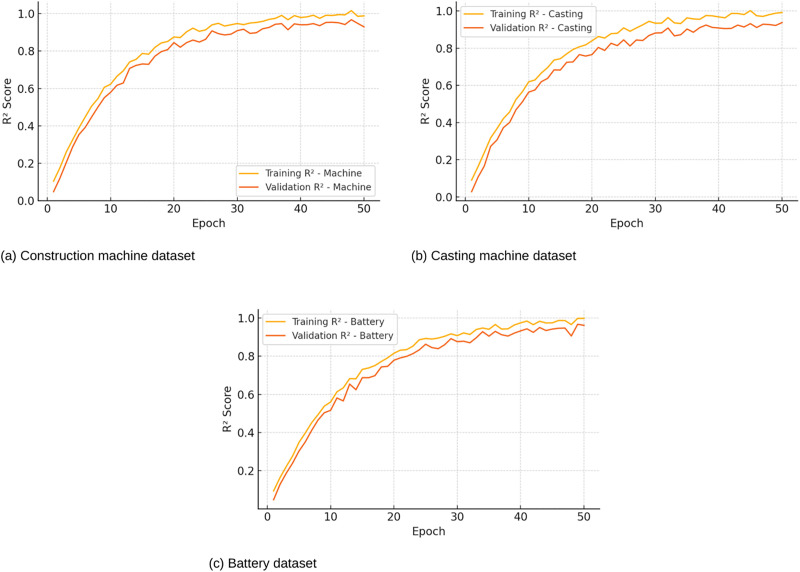
Training and validation R^2^ score curves for the CNN-BiLSTM model across three datasets.

Fig 5(a) shows the R^2^ score progression over training epochs for the construction dataset. Both training and validation curves rise steadily, exceeding 0.85 by epoch 50, which reflects the model’s ability to learn reliable degradation patterns from multivariate mechanical sensor data. Fig 5(b) presents the R^2^ score curves for the casting dataset. Despite the tabular and less sequential nature of the data, the model exhibits consistent learning behavior, achieving validation R^2^ values above 0.80. Fig 5(c) shows the training progress for the battery dataset. The validation R^2^ score steadily improves and peaks near 0.90, confirming the model’s capability to capture long-term electrochemical degradation behavior in battery life forecasting scenarios.

## 5. Discussion

The results of this study demonstrate that the proposed CNN-BiLSTM model provides a robust and generalizable framework for Remaining Useful Life (RUL) prediction across diverse industrial domains. Unlike conventional models that are typically constrained to a single type of equipment or data structure, our approach integrates the strengths of convolutional and recurrent neural architectures to handle both short-term signal variations and long-term degradation trends. The key novelty of this work lies in its cross-domain adaptability, showing that a single unified model can be transferred across mechanical sensor data, metallurgical process parameters, and electrochemical degradation cycles. The zero-shot transfer results reveal the effect of domain shift when no target-domain supervision is used, while the fine-tuned transfer results show that lightweight adaptation with limited labeled target-domain samples can improve predictive performance without modifying the core CNN-BiLSTM feature extractor. This addresses a major gap in the literature, where most prior studies remain narrowly focused on single domains or handcrafted features.

An implicit ablation perspective can be obtained by examining the roles of the individual components in the hybrid architecture. The convolutional layers primarily capture short-term local variations in degradation signals, enabling automatic extraction of localized temporal patterns from raw multivariate inputs. In contrast, the BiLSTM layer models longer-term dependencies by integrating information from both forward and backward temporal directions. Empirical comparisons with standalone recurrent models such as LSTM and GRU, presented in Section 4.3, show that incorporating convolutional feature extraction improves prediction accuracy across the evaluated datasets. This observation highlights the complementary roles of convolutional feature extraction and bidirectional temporal modeling in the proposed framework.

The empirical results highlight several unique strengths of the proposed framework:

- Unified cross-domain design: A single CNN-BiLSTM architecture achieved consistently high accuracy across three heterogeneous datasets, demonstrating scalability and reusability.
- Balanced feature extraction: CNN layers effectively capture local degradation signatures, while BiLSTM layers model long-term dependencies, leading to improved prediction stability.
- Superior accuracy: The proposed method outperformed traditional machine learning and standalone deep learning baselines across MAE, RMSE, and R^2^ metrics, as well as domain-specific Timeliness and PHM08 scores.
- Practical robustness: Training and validation curves indicate stable convergence, suggesting suitability for real-world deployment with limited tuning.
- Cross-domain adaptability: Zero-shot transfer experiments quantify the performance loss caused by domain shift, while fine-tuned transfer experiments show that updating only the fully connected layers using 20% labeled target-domain samples improves adaptation to new industrial domains.

The broader impact of this research is significant. Recent monitoring frameworks such as TFDDP and TFDDCF demonstrate that self-supervised learning can address limited labeled data by extracting informative time–frequency representations from industrial signals [10,11]. In contrast, the proposed CNN–BiLSTM framework operates under a supervised RUL prediction setting and emphasizes temporal degradation modeling, cross-domain transfer, and lightweight fine-tuning. This positions the proposed approach as complementary to self-supervised monitoring models, since time–frequency representation learning can potentially be combined with CNN–BiLSTM sequence modeling for future RUL prediction systems under limited labeled data. By reducing reliance on domain-specific feature engineering, the proposed CNN-BiLSTM framework lowers the entry barrier for predictive maintenance in industries adopting Industry 4.0 practices. Potential applications include:

- Manufacturing: Predictive scheduling of machinery maintenance to minimize downtime and improve throughput.
- Energy systems: Estimating the lifetime of batteries and turbines to enhance grid reliability and optimize replacement planning.
- Transportation: Forecasting wear in engines and critical components of aircraft, vehicles, or rail systems for improved safety and cost efficiency.
- Healthcare devices: Monitoring degradation in medical equipment to ensure reliability in critical care environments.

From a deployment perspective, the proposed CNN–BiLSTM architecture maintains a compact parameter size and moderate computational complexity, as reported in Table 7. Across the three datasets, the total number of trainable parameters ranges from 188,673–192,129, and the estimated FLOPs range from $2.65\times 10^{6}$ to $2.84\times 10^{6}$ for a single forward pass with batch size 1. The measured single-sample inference latency ranges from 0.72 ms to 0.81 ms on an NVIDIA RTX 3090 GPU with 24 GB memory using PyTorch 2.0. These results indicate that the model can support efficient near real-time RUL estimation under the tested GPU environment. Deployment on Jetson-class devices is therefore treated as a feasibility direction rather than a confirmed embedded-hardware benchmark, since latency, memory use, and energy consumption were not directly measured on Jetson devices in this study.

Despite its strengths, several challenges remain:

- Dataset limitations: Current evaluations rely on publicly available datasets that are relatively small and controlled, which may not fully represent the complexity of large-scale industrial systems. In addition, while recent Transformer-based and attention-enhanced architectures have shown promising performance in time-series modeling, they were not included in the current experimental comparison in order to maintain consistent training settings across the heterogeneous datasets. Incorporating such architectures for broader benchmarking represents an important direction for future research.
- Fixed sliding windows: The reliance on fixed-length temporal windows may limit adaptability to variable-length degradation sequences.
- Real-world uncertainties: Issues such as missing sensor data, sensor noise, and concept drift were not explicitly addressed in this study.
- Computational constraints: Although compact, the model may still pose deployment challenges in edge environments with restricted resources.

Building on these findings, several avenues for improvement are identified:

- Attention and transformer-based models: Incorporating attention mechanisms or lightweight transformers could improve adaptability to variable-length sequences and highlight critical degradation intervals.
- Online and continual learning: Developing incremental learning strategies would allow adaptation to evolving industrial environments and mitigate concept drift.
- Uncertainty quantification: Extending the model with probabilistic layers or Bayesian inference could provide confidence intervals, which are valuable for risk-sensitive applications.
- Integration with edge computing: Optimizing and compressing the model would enable deployment on embedded systems for real-time prognostics.
- Broader validation: Testing on more diverse datasets (e.g., aerospace engines, wind turbines, or medical devices) would strengthen evidence of cross-domain generalizability.

Overall, this work demonstrates that a standardized, cross-domain deep learning model can serve as a practical foundation for scalable and transferable prognostic solutions. By bridging gaps between different industrial contexts, the proposed framework advances the development of unified predictive maintenance systems that reduce operational risks and enable data-driven decision-making across sectors.

## 6. Conclusions

This paper presented a unified deep learning framework for Remaining Useful Life prediction using a hybrid CNN-BiLSTM architecture. The proposed model was evaluated across three heterogeneous industrial domains, such as construction machinery, continuous casting machines, and lithium-ion batteries, each with distinct data structures and degradation characteristics. By combining convolutional layers for local pattern extraction with bidirectional LSTM layers for modeling temporal dependencies, the model achieved consistently high predictive accuracy across all datasets. The experimental results demonstrated the model’s superiority over traditional regression methods and standalone deep learning baselines. It achieved the lowest MAE and RMSE scores in all cases and showed strong alignment with domain-specific metrics such as the Timeliness Score and PHM08 Score. Furthermore, the model maintained stable learning behavior, as evidenced by the training and validation curves, and showed cross-domain adaptability through both zero-shot and fine-tuned transfer evaluations. The zero-shot setting assessed direct transfer without target-domain supervision, while the fine-tuned setting used 20% labeled target-domain samples to update only the fully connected layers. These findings support lightweight transfer adaptation under limited target-domain supervision, rather than fully unsupervised cross-domain generalization. The key contribution of this work is the demonstration that a single, reusable architecture can perform reliably across diverse industrial applications without the need for domain-specific tailoring. This has important implications for the scalability and deployment of predictive maintenance solutions in complex operational environments. Future directions include extending the framework to handle online data streams, improving interpretability and uncertainty estimation, and validating the approach on larger-scale and real-time industrial systems. The results in this study lay the groundwork for developing robust, cross-domain prognostic models that reduce downtime, optimize maintenance, and support data-driven operational strategies across industry sectors.
